# Discontinuation rates of different contraceptive methods in Thai women up to 1-year after method initiation

**DOI:** 10.1038/s41598-021-90373-6

**Published:** 2021-05-24

**Authors:** Unnop Jaisamrarn, Monchai Santipap, Somsook Santibenchakul

**Affiliations:** 1 Department of Obstetrics and Gynecology, Faculty of Medicine, Chulalongkorn University, 1873 Rama IV Rd, Pathum Wan, Pathum Wan District, Bangkok, 10330 Thailand; 2 Department of Obstetrics and Gynecology, King Chulalongkorn Memorial Hospital, 1873 Rama IV Rd, Pathum Wan, Pathum Wan District, Bangkok, 10330 Thailand

**Keywords:** Health care, Medical research

## Abstract

We assessed the discontinuation rate and the reason for discontinuation of common contraceptives used by reproductive-aged Thai women. We recruited 1880 women aged 18–45 years from the Family Planning Clinic of the Chulalongkorn Hospital in Bangkok. The participants were followed at three, six and twelve months. A Cox proportional hazards model was used to determine personal risks of discontinuing contraceptives. The incidence rate for discontinuation of combined oral contraceptive pills (COCs), depot medroxyprogesterone acetate (DMPA), copper intrauterine device (IUD), and contraceptive implant(s) were 21.3, 9.2, 4.4, and 2.3/100 person-years, respectively. Most of the women who discontinued (185/222) discontinued contraceptives due to side effects. Compared to contraceptive implant users, the adjusted hazard ratios (aHRs) [95% confidence intervals (CIs)] of discontinuing COCs, DMPA, and the copper IUD were 9.6 (4.3–21.8), 4.2 (1.8–10.0), and 2.2 (0.8–5.9), respectively. Lower income, higher parity, history of miscarriage, and history of abortion were independent predictors of contraceptive discontinuation in a multivariable model.

## Introduction

Although Thailand has been praised as a country that has been highly successful in the implementation of family planning^1^, there is an 8% unmet need for contraception^2^. Since the adoption of the national population policy in 1971, Thailand’s contraceptive use leapt from 14.8% in 1970 to 71.3% in 2019^3^, while the total fertility rate dropped from 4.8 to 1.5^4^. Nevertheless, the incidence of unintended pregnancy and abortion remain reproductive health issues. According to the multiple indicator cluster survey 2019, around 20% of women with a live birth in the last two years stated that their last child was unintended, of which 57.8% were mistimed, and 37.9% were unwanted pregnancy^2^. In 2020, another reproductive health survey conducted by the Ministry of Health revealed that among women who were admitted to the gynecologic unit of public hospitals for termination of pregnancy, half of them stem from unwanted pregnancy^5^. For those who underwent an unsafe abortion, 80% faced serious complications^5^. These pregnancies correlated with adverse pregnancy outcomes and the number of reproductive health consequences^6^. Nonuse or discontinuation of contraception poses a risk factor for these preventable incidents^7^.

Although many studies have been conducted on the discontinuation of contraceptives, most have focused only on a particular group of women or certain contraceptive methods^8^. Thai women are unique in terms of the types of contraceptive methods they commonly use^2^. Existing evidence has demonstrated that the discontinuation of contraception varies according to the type of contraceptive used, age, race, ethnic background, and time when the contraception was initially used^9–11^. For instance, intrauterine devices (IUDs) and contraceptive implant(s) are long-acting reversible contraceptive (LARC) methods and have the lowest discontinuation rate compared to short-acting reversible contraceptives^12^. Younger women tend to discontinue their methods earlier than other age groups^12^. Ethnicity is associated with the rate of contraceptive discontinuation^9^. Evidently, there is a considerable difference in the discontinuation rate of contraception.

Since there is a substantial difference in contraceptive discontinuation among diverse groups, to refine family planning policy in Thailand, public health policy planners need more specific information about reproductive-aged Thai women. Currently, there is limited information regarding the discontinuation of different contraceptives most commonly used by Thai women. Most published studies used retrospective designs and focused on particular contraceptives. Sappan et al. conducted a retrospective chart review of 259 contraceptive implant users and found that 12-month discontinuation was 8.9%^13^. Another retrospective study of 598 etonogestrel contraceptive implant users was conducted by Assavapokee et al. and found that the 12-, 24-, and 36-month discontinuation were 4.6%, 11.6%, and 16.9%, respectively^14^. A small prospective study of 92 contraceptive implant users was conducted by Chaovisitsaree et al. and found that 12-month discontinuation was 7.6%^15^. Unscheduled bleeding was the most common reason for contraceptive implant discontinuation in these studies^13–15^. Chotnopparatpattara et al. conducted a retrospective chart review of 108 adolescents who were depot medroxyprogesterone acetate (DMPA) users and found that 12-month discontinuation was 30.6%^16^. The most common reasons for DMPA discontinuation were unscheduled bleeding, amenorrhea, and weight gain^16^. Nandaa et al. analyzed data from a prospective study of hormonal contraception and human immunodeficiency virus (HIV) acquisition^17^. The authors found that among 486 combined oral contraceptive pills (COCs) and 577 DMPA users, the 12-month discontinuation was 22% and 15%, respectively^17^. Most women reported a personal reason for method discontinuation^17^. Our study aimed to compare discontinuation of different types of four common contraceptives used by Thai. We assessed personal risks of discontinuing various contraceptive methods. The incidence of unintended pregnancy during the one-year period of the study was also examined. This evidence may enable healthcare providers to provide better tailored family planning services to women who are at high risk of discontinuing their use of contraception to prevent unintended pregnancy and abortion.

## Methods

### Study population and procedures

This cohort study enrolled 1912 potentially eligible women who attended the Family Planning and Reproductive Health Clinic, Chulalongkorn Memorial Hospital, Bangkok, Thailand, from January 2009 to December 2012. This period occurred before LARCs became freely available through the national health care program for all adolescents. For this study, the eligibility criteria were as follows: sexually active Thai women aged 18–45 years, not pregnant and with no plans to become pregnant for at least one year after joining the study, desire to initiate one of the four types of contraceptives used in the study [COCs, DMPA, copper IUD, or contraceptive implant(s)], no contraindication for the selected method, and the ability to provide consent in Thai. This study was conducted in accordance with the Declaration of Helsinki. The study was approved by the ethical committee of the Faculty of Medicine, Chulalongkorn University (IRB#162/62). All women signed and dated written informed consent forms approved by the Faculty of Medicine, Chulalongkorn University Institutional Review Board.

### Measurements

All participants underwent standardized contraceptive assessment from the family planning nurses. The following information was acquired from the women: medical history, last menstrual period, history of unprotected sexual intercourse, and history of contraindications to estrogen-progestin contraceptives. General physical examinations, including blood pressure and body weight measurements, were performed. Additionally, if indicated, the women underwent pelvic examination and cervical cytological screening. Subsequently, the women received guidance on up-to-date evidence-based and precise information on each contraceptive method, its effectiveness, risk of side effects and tips on adherence. Then, the women selected their contraceptive method, which was provided by the family planning facility.

At enrollment, all women completed self-administered questionnaires regarding their demographic data. The authors assessed the women’s future pregnancy plan by asking the question, “Do you plan to have children/ any more children some day?” The provided answers were “Yes, I will”, “No, I won’t”, and “I am not sure”. Since sexual intercourse could indirectly affect adherence to contraception, the authors also asked the following question: “How frequently do you have sex?” The choices were either “once a week” or “more than once a week”. All women were followed up to twelve months by interviewing the women either directly at the facility or by telephone call. The women were followed at three, six and twelve months to assess their status of using the contraceptives and the incidence of unintended pregnancies. All women were followed until they discontinued using the contraceptive or completed the study. The authors defined discontinuation of the contraception as abandoning the use of COCs for at least one month, intending to miss a DMPA shot or missing a DMPA shot for at least two weeks, or removal of copper IUD or contraceptive implant(s).

### Statistical analysis

The women’s basic characteristics were reported as the mean, standard deviation, proportion, and percentage. To examine the discontinuation rate among each contraceptive, incidence rate and cumulative incidence based on the Kaplan–Meier (exact-events times) approach were used. A Cox proportional hazards model was used to determine personal risks of discontinuing contraception up to 1 year, and the purposeful selection algorithm was used to decide which covariates should be retained in the final model^18^. Variable age was categorized into adolescent and non-adolescent groups to examine the effect of being adolescents on contraceptive discontinuation^12^. To test the proportional hazards assumption, we plotted the log–log transformation of the survival function for contraceptive type adjusted for other terms in the final multivariable model against the log study time.

## Results

Among the 1912 women who were potentially eligible for this study, we excluded 32 women because they were younger than 18 years old or older than 45 years old. A total of 1880 women were included in this study; 839 (44.6%) women initiated COCs, 494 (26.3%) initiated DMPA, 280 (14.9%) initiated the copper IUD, and 267 (14.2%) initiated contraceptive implant(s). Demographic data with respect to each contraceptive method are shown in Table 1. The answers to two questions, “Do you plan to have children/any more children some day?” and “How frequently do you have sex?”, are also provided in Table 1. Most women had sex more than once a week. Almost all women (99.9%) were cohabiting or married.

**Table 1:** Baseline characteristics of the women and their use of the contraceptive method (N = 1880).

Independent variable	COCs^a^ n = 839	DMPA^b^ n = 494	IUD^c^ n = 280	Implant(s) n = 267	Total N = 1880
**Age, years, n (%)**					
18–19	21 (2.5)	25 (5.1)	4 (1.4)	19 (7.1)	69 (3.7)
20+	818 (97.5)	469 (94.9)	276 (98.6)	248 (92.9)	1811 (96.3)
**BMI**^**d**^**, n (%)**^21^					
< 18.5	80 (9.6)	35 (7.1)	43 (15.3)	16 (6.0)	174 (9.3)
18.5–22.9	643 (76.6)	437 (88.5)	234 (83.6)	247 (92.5)	1561 (83.0)
23.0–24.9	53 (6.3)	18 (3.6)	3 (1.1)	4 (1.5)	78 (4.1)
≥ 25.0	63 (7.5)	4 (0.8)	0	0	67 (3.6)
**Occupation, n (%)**					
Employee	503 (60.0)	259 (52.5)	198 (70.7)	143 (53.6)	1103 (58.7)
Housewife	204 (24.3)	136 (27.5)	54 (19.3)	66 (24.7)	460 (24.4)
Others	132 (15.7)	99 (20.0)	28 (10.0)	58 (21.7)	317 (16.9)
**Income, baht**^**e**^**, n (%)**					
≤ 9000	279 (32.9)	151 (30.6)	72 (25.7)	76 (28.5)	575 (30.6)
> 9000	563 (67.1)	343 (69.4)	208 (74.3)	191 (71.5)	1,305 (69.4)
**Religion, n (%)**					
Buddhist	831 (99.1)	486 (98.4)	277 (98.9)	265 (99.2)	1859 (98.9)
Christian	2 (0.2)	1 (0.2)	0	0	3 (0.2)
Muslim	6 (0.7)	7 (1.4)	3 (1.1)	2 (0.8)	18 (0.9)
**Parity, n (%)**					
0	14 (1.7)	0	4 (1.4)	1 (0.4)	19 (1.0)
1–2	807 (96.2)	481 (97.4)	273 (97.5)	249 (93.2)	1810 (96.3)
≥ 3	18 (2.1)	13 (2.6)	3 (1.1)	17 (6.4)	51 (2.7)
**History of miscarriage or abortion, n (%)**					
Yes	176 (21.0)	77 (15.6)	30 (10.7)	40 (15.0)	301 (16.0)
No	663 (79.0)	417 (84.4)	250 (89.3)	227 (85.0)	1579 (84.0)
**Do you plan to have children/any more children some day? n (%)**					
A. Yes	319 (38.0)	147 (29.8)	63 (22.5)	76 (28.5)	(2.2)
B. No	237 (28.3)	166 (33.6)	111 (39.6)	106 (39.7)	(3.0)
C. Not sure	283 (33.7)	181 (36.6)	106 (37.9)	85 (31.8)	655 (34.8)
**How frequently do you have sex?, n (%)**					
A. Once a week	177 (21.1)	119 (24.1)	73 (26.1)	54 (20.2)	2.5)
B. More than once a week	662 (78.9)	375 (75.9)	207 (73.9)	213 (79.8)	1457 (77.5)

^a^COCs: Combined oral contraceptive pills.

^b^DMPA: Depot medroxyprogesterone acetate.

^c^IUD: Copper intrauterine device.

^d^According to the Asian BMI category, < 18.5 was defined as underweight, 18.5–22.9 was defined as normal weight, 23.0–24.9 was defined as overweight, and ≥ 25.0 was defined as obese^18^.

^e^1 US dollar equals 31.25 Thai baht (2012).

**Figure 1:**
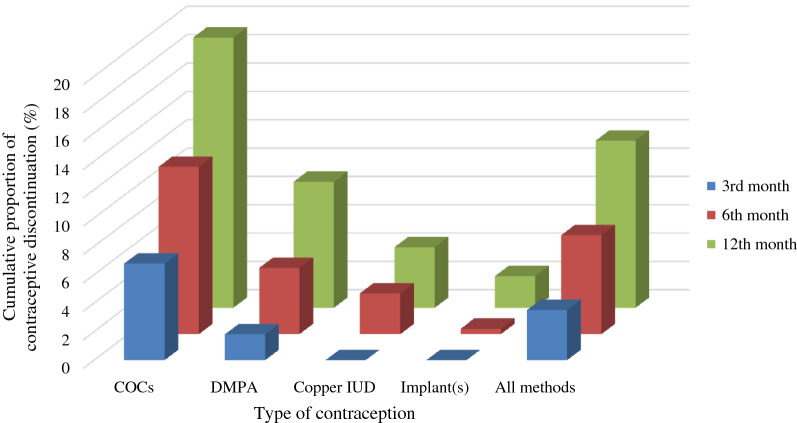
Cumulative proportion of contraceptive discontinuation at 3, 6 and 12 months.

**Figure 2:**
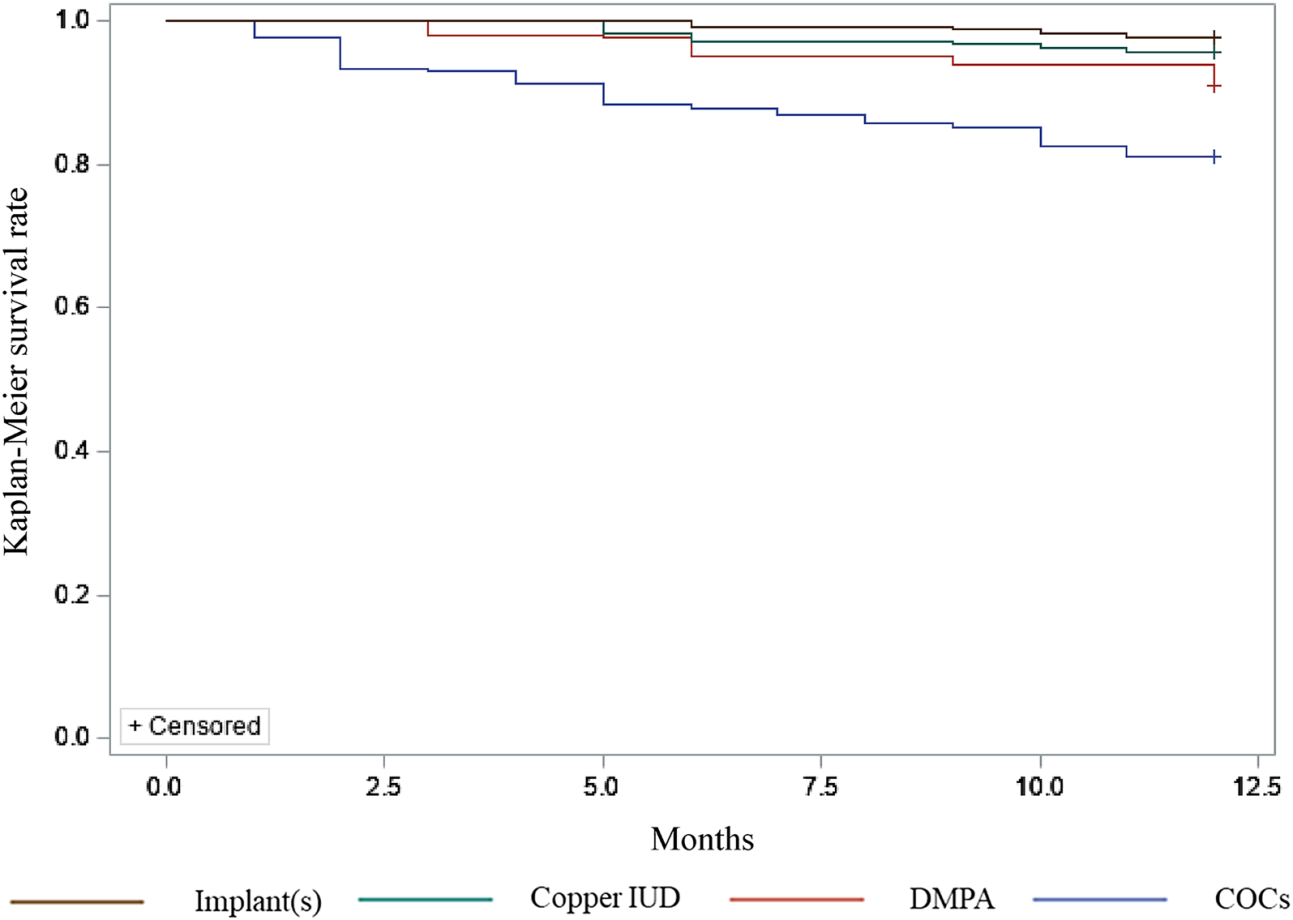
Kaplan–Meier survival rate of each contraceptive. From top to bottom, the top line represents contraceptive implant(s), the line below that represents copper IUD, the third line represents DMPA and the bottom line represents COCs.

The cumulative probability of discontinuation for each contraceptive and all methods at three, six, and twelve months are shown in Fig. 1. Copper IUDs and contraceptive implant(s), considered LARCs, had low cumulative discontinuation rates during the 1-year period. None of the participants were lost to follow-up. The incidence rate for discontinuation of COCs was 21.3/100 person-years, which had the highest rate of discontinuation among all four methods. The incidence rate for discontinuation of DMPA was 9.2/100 person-years. Among the LARC group, the incidence rate for discontinuation of copper IUD was 4.4/100 person-years. The incidence rate for discontinuation of contraceptive implant(s) was 2.3/100 person-years, which was the lowest among all four methods. The survival probabilities of each contraceptive during the one-year period are shown in Fig. 2. The distribution of the main reason for the discontinuation of each contraceptive is shown in Table 2. Most women (185/222) discontinued their contraceptives because of side effects. The most common side effects for COC discontinuation were nausea/vomiting (51/126) and headache (42/126). For DMPA, bleeding and spotting (35/41) were the major causes of discontinuation. For the copper IUD, pelvic pain and dysmenorrhea (10/12) were the main causes of discontinuation. For contraceptive implant(s), all women (6/6) stated that they discontinued their contraceptive implant(s) due to spotting. Among all women who discontinued their contraceptives, nine women had unintended pregnancy that ended in induced abortion. All of these women discontinued COCs.

**Table 2:** Distribution of the main reason for discontinuation by contraceptive (n = 222).

Reasons	COCs^a^	DMPA^b^	IUD^c^	Implant(s)	Total
Side effects, n (%)	126 (78.8)	41 (93.2)	12 (100)	6 (100)	185 (83.3)
Wanted to become pregnant, n (%)	4 (2.5)	3 (6.8)	0	0	7 (3.1)
Cumbersome to take the pills, n (%)	29 (18.1)	0	0	0	29 (13.1)
Ended her relationship, n (%)	1 (0.6)	0	0	0	1 (0.5)
Number of discontinuations, n (%)	160 (100)	44 (100)	12 (100)	6 (100)	222 (100)

^a^COCs: Combined oral contraceptive pills.

^b^DMPA: Depot medroxyprogesterone acetate.

^c^IUD: Copper intrauterine device.

The unadjusted hazard ratio (HR) and adjusted hazard ratio (aHR) with a 95% confidence interval (CI) are shown in Table 3. The results show that the type of contraceptive, income level, parity, history of miscarriage and history of abortion were predictors of contraceptive discontinuation in the final Cox proportional hazards model. In addition, COC use, history of abortion, and DMPA use were the top three predictors of 1-year contraceptive discontinuation; compare to contraceptive implant users, the aHR of using COCs was as high as 9.6 (4.3–21.8); compare to users without a history of abortion, users with a history of abortion had an aHR equal to 5.1 (3.0–8.8); and compare to contraceptive implant users, DMPA use had an aHR of 4.2 (1.8–10.0). The proportional hazards assumption was met as the log–log plots showed curves that were approximately parallel.

**Table 3:** The unadjusted and adjusted hazard ratios from the Cox proportional hazards model.

	Unadjusted model		Adjusted model	
	HR^a^	(95% CI^b^)	aHR^c,d^	(95% CI^b^)
**Type of contraceptive**				
COCs^e^	9.4	4.2–21.2	9.6	4.3–21.8
DMPA^f^	4.1	1.7–9.5	4.2	1.8–10.0
IUD^g^	1.9	0.7–5.1	2.2	0.8–5.9
Implant(s)	Reference		Reference	
**Age (years)**				
18–19	1.4	0.8–2.6		
20 +	Reference			
**BMI**^**h**^ **(kg/m**^**2**^**)**^21^				
< 18.5	1.00	0.6–1.6		
18.5–22.9	Reference			
23.0–24.9	2.2	1.4–3.6		
≥ 25.0	2.0	1.2–3.5		
**Income (Baht**^**i**^**)**				
≤ 9000	1.6	1.2–2.1	1.5	1.2–2.0
> 9000	Reference		Reference	
**Career**				
Employee	Reference			
Housewife	1.3	0.9–1.7		
Others	0.8	0.5–1.2		
**Parity**				
0	1.5	0.5–4.6	0.7	0.2–2.1
1, 2	Reference		Reference	
≥ 3	2.1	1.2–3.8	2.5	1.4–4.5
**History of miscarriage**				
Yes	1.7	1.3–2.2	1.6	1.2–2.2
No	Reference		Reference	
**History of abortion**				
Yes	5.6	3.3–9.5	5.1	3.0–8.8
No	Reference		Reference	
**Do you plan on having children someday?**				
A. Yes	0.8	0.6–1.1		
B. No	Reference			
C. Not sure	0.7	0.5–0.9		
**How frequently do you have sex?, n, %**				
A. Once a week	Reference			
B. More than once a week	0.7	0.5–0.9		

^a^HR: Hazard ratio.

^b^CI: Confidence interval.

^c^aHR: Adjusted hazard ratio.

^d^Adjusted for type of contraceptive, income, parity, history of miscarriage, and history of abortion.

^e^COCs: Combined oral contraceptive pills.

^f^DMPA: Depot medroxyprogesterone acetate.

^g^IUD: Copper intrauterine device.

^h^According to the Asian BMI category, < 18.5 was defined as underweight, 18.5–22.9 was defined as normal weight, 23.0–24.9 was defined as overweight, and ≥ 25.0 was defined as obese^18^.

^i^1 US dollar equals 31.25 Thai baht (2012).

## Discussion

Among all four types of contraceptives, COCs were the most popular type used by Thai women and had the highest discontinuation rate during the 1-year period of use. DMPA had a lower discontinuation rate than COCs but a higher discontinuation rate than the copper IUD and contraceptive implant(s). Contraceptive method choice was associated with risk of discontinuation, with evidence that users of COCs and DMPA have higher risk of discontinuation. These results were consistent with previous clinical studies^12, 19^, which showed lower contraceptive discontinuation among women who used LARC compared to other methods. However, the rates of contraceptive discontinuation during the 1-year period for all four types of contraceptives in this study were quite low compared to other studies^13–17^. One of the possible explanations for this is that some women might be former users of the selected method since we included contraceptive initiation during postpartum visits. Additionally, we included only women who had no plans to become pregnant for at least one year, contributing to the lower rate of contraceptive discontinuation. This study was conducted at a specialized family planning facility located in a tertiary care setting that provided extended counseling and comprehensive family planning services. Therefore, it should be noted that the results of this study cannot be generalized to other clinical settings that do not provide such services.

The side effects of each contraceptive method played a major role in contraceptive discontinuation. These adverse effects are predictable and specific to each contraceptive. For instance, nausea/vomiting, headache, and breast tenderness, which are associated with estrogen, are commonly experienced by COC users. Similarly, progestin was associated with bleeding and spotting among DMPA and contraceptive implant(s) users. These bothersome side effects usually occur during the first few months of contraceptive use and can be mitigated by appropriate medical management. Hence, to encourage the continuation of the contraceptive, family planning providers should tailor counseling according to the type of contraceptive a woman has selected and inform her about the side effects. In addition, the cumbersome side effects of the contraceptive should be managed properly. For example, women who complain of unscheduled bleeding from progestin contraceptive implants should be evaluated to exclude gynecologic causes. Their concerns should be assessed and reassured. We prescribed short-course estrogen therapy to women whose bleeding interfere with their normal daily activities.

Lower income level, higher parity, and history of abortion and miscarriage were considered predictors for 1-year contraceptive discontinuation in this study. Lower income and higher parity were also predictors of contraceptive discontinuation in another clinical study^20^. A lower income level might be associated with low education, so it is possible that women may not understand the importance of using contraceptives to prevent unintended pregnancy^19^. Unfortunately, this study did not include information regarding education level. For history of abortion, this independent variable showed the second highest hazard ratio (aHR 5.1, 95% CI 3.0–8.8). Since abortion stems from unintended pregnancy, this might imply that a history of unintended pregnancy is also a predictor of contraceptive discontinuation.

This study has some limitations. Some independent variables, such as the intention to have children and frequency of sexual activity, were assessed only once at the onset of the study, although these factors tended to change over time. Therefore, the association between these variables and contraceptive discontinuation might contain some information bias.

Aside from the limitations, the strength of this study was the large sample size, and none of the women were lost to follow-up. This study assessed the discontinuation of four commonly used contraceptives among healthy reproductive-aged Thai women. This information is valuable for family planning facilities and provides strategies to ensure that contraceptives are used continuously until there is a desire to start a family. Women should be provided with tailored comprehensive family planning counseling and services to mitigate the issue of early contraceptive discontinuation.

## Conclusion

Among Thai women, LARC users were associated with a lower rate of 1-year contraceptive discontinuation. Women with lower income, higher parity, history of miscarriage and abortion had higher risks for contraceptive discontinuation. Family planning providers should seek appropriate strategies to mitigate the issue of contraceptive discontinuation. The primary reason for discontinuing use of the various contraceptive methods among Thai women up to 1 year after method initiation was side effects.
